# High incidence of triple negative breast cancers following pregnancy and an associated gene expression signature

**DOI:** 10.1186/s40064-015-1512-7

**Published:** 2015-11-19

**Authors:** Szilard Asztalos, Thao N. Pham, Peter H. Gann, Meghan K. Hayes, Ryan Deaton, Elizabeth L. Wiley, Rajyasree Emmadi, Andre Kajdacsy-Balla, Nilanjana Banerji, William McDonald, Seema A. Khan, Debra A. Tonetti

**Affiliations:** Department of Biopharmaceutical Sciences, University of Illinois at Chicago, Chicago, IL USA; Department of Pathology, University of Illinois at Chicago, Chicago, IL USA; Allina Health, Minneapolis, MN USA; Department of Surgery, Northwestern Feinberg School of Medicine, Chicago, IL USA

**Keywords:** Pregnancy-associated breast cancer, Parity, Triple negative breast cancer, Inflammation

## Abstract

Breast cancer risk increases transiently in the period following pregnancy; pregnancy-associated breast cancers (PABC) are more aggressive than cases diagnosed in nulliparous women. We have previously reported that in the normal human breast pregnancy results in the upregulation of a number of inflammation related genes, suggesting a pro-tumorigenic environment as well as downregulation of *ESR1* (*ERα*) and *ERBB2* (*HER2*) and upregulation of *ESR2* (*ERβ*), suggesting a protective effect. In this study, we aimed to investigate the possibility of differential regulation of the same gene set modulated in the normal breast, in human breast tumors following pregnancy. Gene expression was measured by real-time PCR on tumor regions isolated by laser capture microdissection from paraffin sections. Immunohistochemistry was performed on tissue microarrays (TMA) for protein expression. Hierarchical clustering was performed using the average linkage method to determine coordinate expression of sets of genes. We find that breast cancers detected within 10 years following pregnancy display a different gene expression pattern than those detected in nulliparous breast cancer patients. The gene expression difference is mainly attributable to a triple negative (TNBC) subgroup found to be more frequent in PABCs up to 10 years following a pregnancy. We also show that protein and mRNA expression levels correlate in half of the proteins tested by TMA. Despite the fact that this is a small study of 53 patients, we identified a gene expression signature that is differentially expressed in pregnancy-associated TNBC.

**Electronic supplementary material:**

The online version of this article (doi:10.1186/s40064-015-1512-7) contains supplementary material, which is available to authorized users.

## Background

Epidemiological studies have long established a link between breast cancer risk and a completed pregnancy. Pregnancy, especially if it occurs at an early age, is generally considered to be protective against breast cancer. However, rigorous epidemiological studies have shown that the protection conferred by pregnancy is delayed and that the period following completion of pregnancy/lactation is characterized by an increase in breast cancer risk (Schedin 2006; Albrektsen et al. 2005; Lambe et al. 1994). This risk period encompasses at least 7–10 years, and varies according to, among other factors, the age of the mother at first pregnancy, with older first time mothers showing the longest increased risk period. Pregnancy-associated breast cancers (PABCs) diagnosed within this period tend to be aggressive, with high mortality rates (Bladstrom et al. 2003; Johansson et al. 2011). In women diagnosed with breast cancer within 1 year of giving birth, the 15 year survival rate is only half that of age matched nulliparous women (Whiteman et al. 2004).

One hypothesis, supported by animal model data, asserts that the process of breast involution following pregnancy is tumor-promoting and/or tumorigenic, due to the profound remodeling that occurs in the mammary gland during this time (Schedin 2006; Lyons et al. 2011; Clarkson et al. 2004). These changes include extracellular matrix (ECM) remodeling, angiogenesis and inflammatory processes that share characteristics with wound healing programs as evidenced by animal studies of forced weaning (Stein et al. 2004).

By utilizing a gene set containing 64 genes involved in inflammation, ECM remodeling, angiogenesis and a number of breast cancer biomarkers, we have previously reported that the normal human breast environment following pregnancy is associated with upregulation of a number of inflammation related genes (Asztalos et al. 2010), supporting the hypothesis for the role of involution in PABC. At the same time we found evidence for a protective effect, as shown by repression of *ESR1* (ERα) and *ERBB2* (HER2-neu) and increased expression of *ESR2* (ERβ). In the present study we investigated the possibility of differential regulation of the same set of genes in human breast tumors from nulliparous and parous patients. We find that breast tumors detected following a pregnancy show a different gene expression pattern than those detected in nulliparous women. The gene expression difference is mainly attributable to a triple negative breast cancer (TNBC) subgroup that was more prevalent in PABCs than in the nulliparous group. This parous TNBC subgroup was characterized by differential expression of 14 out of the 64 genes, compared to nulliparous subjects and parous non-TNBC subjects, assayed in the study. While our findings independently confirm other studies that report a higher incidence of TNBC diagnosed in PABCs compared to nulliparous patients (Pilewskie et al. 2012; Madaras et al. 2014), this study is the first to report the prevalence of TNBC in patients up to 10 years post-pregnancy and its association with a TNBC-specific gene set.

## Methods

### Patients and samples

Patients between 18 to 45 years of age who were diagnosed with breast cancer, selected from the University of Illinois at Chicago Hospital, Northwestern Memorial Hospital (Chicago) and Abbott Northwestern Hospital (Minneapolis). The Institutional Review Boards of each institution approved this study (Protocol #2006-0889). Patients were eligible for study if their paraffin blocks were available and parity status and time interval since their last pregnancy was known. Patients were divided into categories according to the time elapsed since their last pregnancy at the time of tumor tissue collection as follows: nulliparous, recent pregnancy (0–2 years since last pregnancy) and more distant pregnancy (5–10 years since pregnancy). The clinical characteristics of the tumors and patients are summarized in Table 1.

**Table 1:** Sample characteristics

	Nulliparous	Recent pregnancy	Distant pregnancy
Sample size	19	17	17
Tumor size (cm) (mean ± SD)	2.7 ± 1.7	2.0 ± 1.2	4.6 ± 4.5
Age at diagnosis (mean ± SD)	36 ± 5	38 ± 3	36 ± 7
Pregnancies (mean ± SD)	0	2.0 ± 1.0	1.9 ± 0.7
Tumor grade			
I	2 (11 %)	0 (0 %)	2 (12 %)
II	12 (63 %)	2 (12 %)	4 (24 %)
III	4 (21 %)	13 (76 %)	10 (59 %)
NA	1 (5 %)	2 (12 %)	1 (6 %)
ERα+	16 (84 %)	9 (53 %)	7 (41 %)
PR+	10 (53 %)	9 (53 %)	6 (35 %)
Her2/neu+	8 (42 %)	6 (35 %)	5 (29 %)
Triple negative status			
TNBCs	1 (5 %)	7 (41 %)	7 (41 %)
Non-TNBCs	18 (95 %)	10 (59 %)	10 (59 %)

Unless otherwise stated, numbers indicate patients in each category with percent contribution in parentheses

### Laser capture microdissection, RNA isolation, cDNA synthesis, linear amplification

Laser capture microdissection of tumor regions from paraffin sections was done as previously described (Asztalos et al. 2010). RNA was extracted from 19 nulliparous, 17 recent pregnancy and 17 distant pregnancy samples. RNA isolation, cDNA synthesis and pre-amplification was done as previously described (Asztalos et al. 2010).

### Real time PCR

We selected 64 genes involved in the processes of inflammation, ECM remodeling or angiogenesis, as previously described (Asztalos et al. 2010). Gene expression was measured using customized Taqman^®^ assays that amplified short amplicons. For each sample, C_t_ values of each gene of interest were normalized to the average C_t_ values of housekeeping genes *ACTB* and *HPRT1* (delta C_t_). Delta C_t_ values (or housekeeping-gene adjusted gene expression) were used for statistical analysis for differences among groups (Additional file 1). For the ease of presenting these differences, delta C_t_ values were anti-logged and expressed as fold-changes relative to a reference group within the study population.

### Immunohistochemistry (IHC)

TMA blocks were sectioned to 4 µm thickness. IHC for ERα, PR and HER2 was performed at the University of Illinois at Chicago Medical Center following standard protocols with a Ventana Benchmark^®^ system. For all other proteins, following deparaffinization/rehydration, samples were incubated in sodium citrate antigen retrieval buffer pH 6.0 at 95 °C for 20 min and allowed to cool to room temperature. IHC was then performed using the Dako Envision Plus^®^ system. Antibodies, incubation times and dilutions used can be found in Additional file 2. Slides were counterstained with hematoxylin and permanently mounted.

### Tissue microarrays (TMAs) and image analysis

TMAs were constructed using 2 mm diameter cores. For each patient, triplicate cores were placed adjacent to each other on the TMA; samples belonging to the three pregnancy categories were placed at random to avoid positional effects. After staining, samples were scanned at 200× using an Aperio ScanScope CS^®^ (Leica Biosystems, Inc., Vista, CA) digital microscopy system. Tumor regions were outlined using the software draw tool. The various stains were analyzed by automated Aperio algorithms, according to their predominant pattern of localization within cells. ERα and PR were scored by a nuclear algorithm; HER2 and CDH1 by a membrane algorithm; and CXCL1, TGFB3 by an algorithm for cytoplasmic staining. Calculations for nuclear and membrane algorithm can be found in Additional file 3. ER, PR and HER2 clinical status was provided by each hospital and was independently verified by co-investigator pathologists (RE, EW) and by digital image analysis. In cases of disagreement between pathologist and digital analysis score, the pathologist score was used; however, there was excellent correlation between the two scoring methods (*p* < .001).

### Statistical analysis

Expression of individual genes was compared between groups with either a two-sided *t* test, when two groups were compared, or a one-way ANOVA, followed by Tukey’s HSD test, when more than two groups were compared. Unsupervised hierarchical clustering was performed using the average linkage method to determine coordinate expression of sets of genes (Cluster software) (Eisen et al. 1998), and results were visualized with Treeview (Eisen et al. 1998). To analyze distribution of TNBC samples between nulliparous and parous groups, Fisher’s exact test was used.

For TMA data analysis, triplicate spots (where available—some spots were excluded due to missing tissue) were averaged. Statistical analysis of normally distributed data consisted of one-way ANOVA, followed by Tukey’s HSD test for three groups or a two-sided *t* test for two groups. For non-normally distributed data, Wilcoxon’s exact test was used.

## Results

### Prevalence of high grade, triple negative subtype in parous samples

Our patient population was closely matched for age at diagnosis and average tumor size (Table 1). We noticed that the two parous groups displayed more tumors of higher grade compared to the nulliparous group. Indeed, there is a significant difference in terms of grade distribution when the nulliparous group was compared to recent pregnancy (*p* < 0.001), distant pregnancy (*p* < 0.05), and the combined parous group (*p* < 0.001) respectively. We also found that this combined parous group presented a higher percentage of TNBC cases compared to the nulliparous group (Fisher’s exact test, *p* < 0.01). The incidence of HER2-expressing cases observed in all groups is higher than for an unselected breast cancer population (~20 %), which reflects the limitation of our small sample size.

### Identification of genes differentially expressed between tumors of nulliparous and parous women

Since it is reported that breast cancers occurring at various time intervals following pregnancy differ in terms of their outcomes (Schedin 2006), we initially distinguished between a recent pregnancy and distant pregnancy group. However, since we found that no genes were differentially expressed between the recent (<2 years) and distant parity (5–10 years) groups (Table 2), we combined them into a single parous group for subsequent analyses. Eight genes are differentially expressed in breast cancer tissues when the parous and nulliparous subjects are compared, as summarized in Table 2.

**Table 2:** Genes showing differential expression between nulliparous and parous tumors as determined by real time PCR

	Nulliparous	Recent pregnancy	Distant pregnancy	Parous	*p* value
*CXCL1*	1 ± 0.2	4.6 ± 1.1	4.7 ± 1.5	4.4 ± 1.6	0.005
*THBS1*	1 ± 0.2	0.5 ± 0.0	0.5 ± 0.1	0.5 ± 0.0	0.01
*ESR1*	1 ± 0.2	0.3 ± 0.0	0.2 ± 0.0	0.3 ± 0.1	0.01
*ELN*	1 ± 0.2	0.4 ± 0.0	0.4 ± 0.0	0.4 ± 0.1	0.018
*TGFB3*	1 ± 0.1	0.5 ± 0.0	0.5 ± 0.0	0.6 ± 0.1	0.022
*ADAM9*	1 ± 0.1	0.6 ± 0.0	0.6 ± 0.0	0.6 ± 0.0	0.025
*IL11*	1 ± 0.1	0.5 ± 0.0	0.5 ± 0.0	0.5 ± 0.1	0.026
*CDH1*	1 ± 0.1	0.5 ± 0.0	0.6 ± 0.0	0.5 ± 0.0	0.037

Gene expression averages were normalized to the nulliparous group. Mean ± SE are shown. Parous includes recent and distant pregnancies. *p* value is for *t* test comparing nulliparous to parous. N nulliparous tumors = 19, N recent pregnancy tumors = 17, N distant pregnancy tumors = 17, N parous (combined recent and distant pregnancy) = 34

**Fig. 1:**
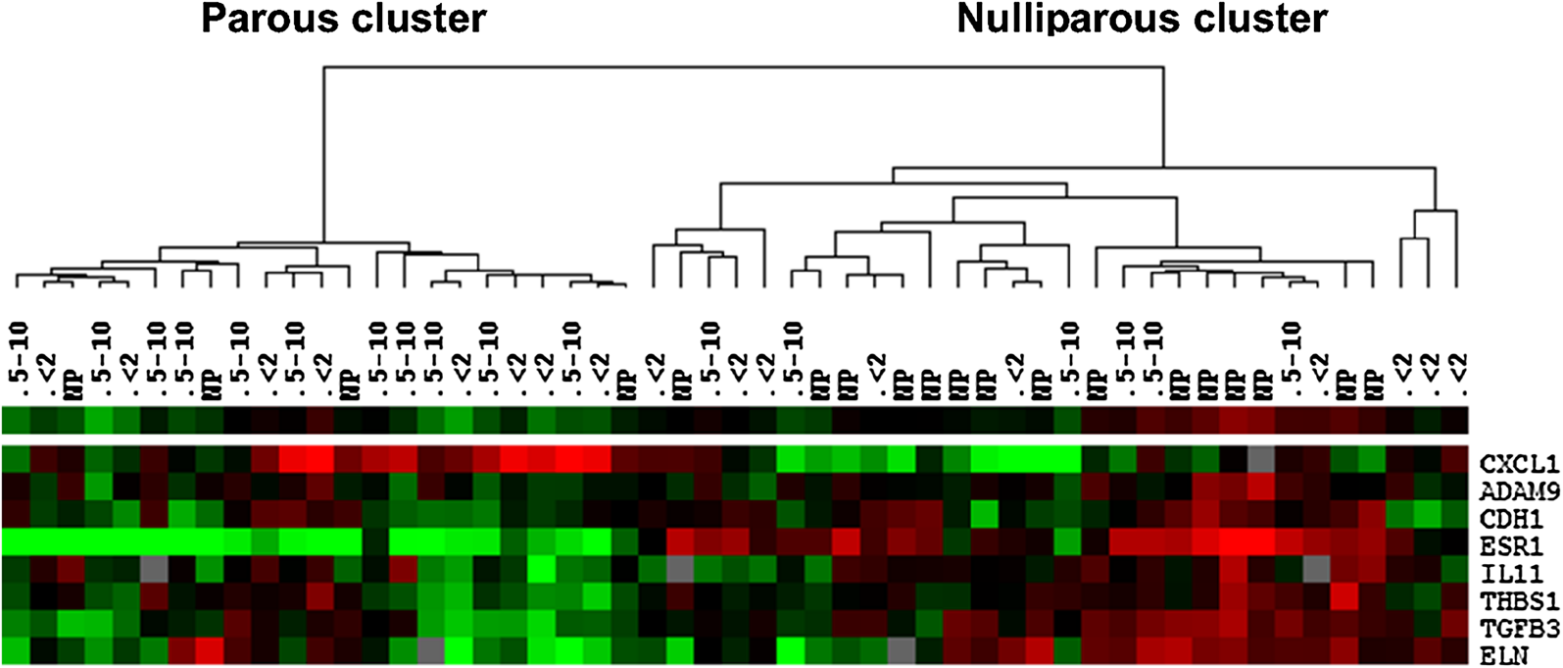
Clustering of tumor samples based on the genes significantly differently expressed between the nulliparous (NP) and parous (P) tumor samples. N NP = 19, N P = 34

We performed an unsupervised clustering analysis to determine the ability of this eight-gene set to differentiate nulliparous and parous tumors. The hierarchical clustering tree is shown in Fig. 1. Based on the first bifurcation, one cluster is predominantly composed of parous subjects and the other of nulliparous subjects; 56 % of parous subjects were included in the “parous” cluster, versus only 21 % of the nulliparous subjects (Fisher’s exact, *p* = 0.02). Correspondingly, 79 % of the nulliparous patients and 44 % of the parous patients were included in the “nulliparous cluster”. This suggests that the nulliparous group was somewhat more homogenous in terms of the expression of these eight genes than the parous group.

### Prevalence of TNBC by parity group and a triple negative gene set

Given the high frequency of TNBCs in the parous groups compared to the nulliparous as mentioned above, we were interested in determining whether this group is characterized by a different gene expression profile. We therefore separated our samples into three groups: parous TNBC, parous non-TNBC, and nulliparous non-TNBC. The nulliparous TNBC samples were not included as a group since we only had one sample in that category. Of the 64 total genes, 14 genes characterized the parous TNBCs (Table 3). These 14 genes included 5 of the 8 genes found to be associated with parous breast cancers (*CXCL1*, *THBS1*, *ESR1*, *ELN*, *TGFB3*) (Table 2), which were found to be down-regulated in the parous TNBC group compared to the other two. This suggests that the differences originally identified between the nulliparous and parous cancer groups were potentially due to the parous TNBC subgroup. It is worthwhile to note that none of these 14 genes was found to be different between the non-TNBC parous and non-TNBC nulliparous groups.

**Table 3:** Genes differentially expressed between parous TNBCs, parous non-TNBCs and nulliparous non-TNBCs

	Parous TNBCs (A)	Parous non-TNBCs (B)	Nulliparous non-TNBCs (C)	A versus B	A versus C	B versus C
*CXCL1*	1.0 ± 0.3	0.3 ± 0.1	0.1 ± 0.0	*	***	ns
*CXCL12*	1.0 ± 0.2	3.4 ± 0.5	2.3 ± 0.3	***	*	ns
*ELN*	1.0 ± 0.4	2.2 ± 0.4	3.3 ± 0.7	**	**	ns
*ERBB2*	1.0 ± 0.1	25.8 ± 8.4	5.6 ± 1.1	***	***	ns
*ESR1*	1.0 ± 0.3	23.7 ± 5.1	49.9 ± 14.4	***	***	ns
*FBN1*	1.0 ± 0.3	2.1 ± 0.3	2.5 ± 0.5	**	**	ns
*IL1A*	1.0 ± 0.7	0.1 ± 0.0	0.1 ± 0.0	*	**	ns
*IL8*	1.0 ± 0.3	0.4 ± 0.1	0.4 ± 0.2	**	**	ns
*MMP12*	1.0 ± 0.5	0.2 ± 0.1	0.4 ± 0.2	*	*	ns
*MMP2*	1.0 ± 0.3	1.9 ± 0.3	2.0 ± 0.4	*	*	ns
*PGR*	1.0 ± 0.3	154.1 ± 75.4	162.8 ± 57.0	***	***	ns
*TGFB3*	1.0 ± 0.2	3.3 ± 0.4	4.2 ± 0.7	***	***	ns
*THBS1*	1.0 ± 0.3	1.8 ± 0.2	2.6 ± 0.6	***	***	ns
*TIMP2*	1.0 ± 0.3	2.1 ± 0.3	2.0 ± 0.3	***	**	ns

Statistical analysis was done using delta C_t_ values or housekeeping-gene adjusted gene expression (see Additional file 1). For the ease of presenting these differences, delta C_t_ values were anti-logged and expressed as fold-changes relative to parous TNBC group (A) as seen in the table below. All genes were significantly differently expressed in the parous TNBC group compared to the other two groups based on ANOVA followed by Tukey’s HSD test. The values for the parous (B) and nulliparous non-TNBCs (C) were not significantly different from each other. TNBC nulliparous group was not included since it contains only one sample. CT values were normalized to parous TNBC group (A). Averages ± SE shown

*ns* not significant

For Tukey’s HSD test * *p* < 0.05; ** *p* < 0.01; *** *p* < 0.001 significant levels

**Fig. 2:**
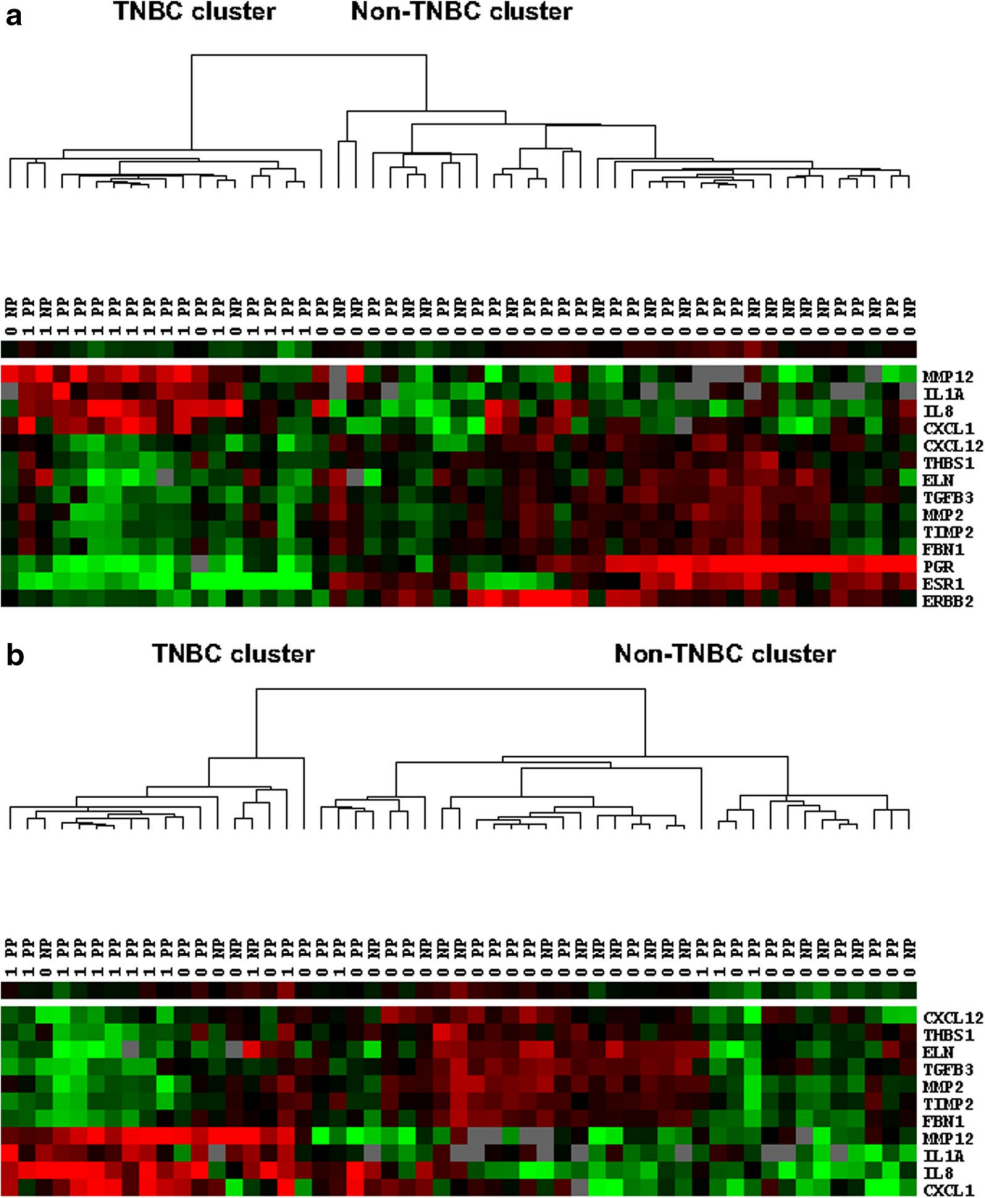
Clustering of tumor samples based on the gene set of **a** 14 genes including *ESR1*, *PGR*, and *ERBB2*, and **b** 11 genes excluding *ESR1*, *PGR*, and *ERBB2.* Samples labeled with 1 are TNBCs, samples labeled with 0 are non-TNBCs. *NP* nulliparous, *PP* parous, N TN parous = 14, N non-TN parous = 20, N TN nulliparous = 1, N non-TN nulliparous = 18

To determine how these 14 genes (Table 3) can differentiate between TNBCs and non-TNBCs, average linkage clustering was performed. Judged by the first bifurcation, the clustering power of the gene set was very efficient, with only four of 38 non-TNBC samples and none of 15 TNBC samples misclassified (Fig. 2a). Since three of these 14 genes (*ESR1*, *PGR*, and *ERBB2*) themselves identify the TNBC subtype, we repeated average linkage clustering with the other 11 genes and examined their efficiency at differentiating TNBC from non-TNBC. We observed that even without these three biomarkers, the gene set was able to correctly classify 11 out of 15 TNBC cases (Fig. 2b).

### IHC and correlation between mRNA and protein levels for selected genes

Given that mRNA and protein levels do not necessarily correlate, we were interested in the identification of possible protein level differences between the parous and nulliparous cancers. TMAs were constructed and IHC was performed for six proteins. These proteins were chosen because they were part of either the 8- or 14-gene set set identified in Tables 2 and 3 (ERα, PR, HER2, CDH1, CXCL1, TGFB3). Since no differences in protein expression were detected between the two parous categories, they were again pooled into one parous group and compared to the nulliparous category. We observed a statistically significant correlation between mRNA and protein level for ERα, PR and HER2, with correlation coefficients of 0.71, 0.70, and 0.79 (all *p* < 0.0001) respectively. CDH1 mRNA and protein expression were also statistically correlated, albeit less strongly (correlation coefficient 0.4, *p* = 0.01). We found no correlation between mRNA and protein level for CXCL1 and TGFB3 Additional file 4.

## Discussion

It is now recognized that the post pregnancy period is associated with an increased breast cancer incidence and more aggressive breast cancers (Schedin 2006; Ali et al. 2012; Lyons et al. 2009). It is hypothesized that a variety of factors are responsible for these findings, including the hormonal milieu, immune suppression during pregnancy, as well as difficulties in detection of cancers following birth due to increased breast density (Schedin 2006; Lyons et al. 2009). Involution, the natural physiological process by which the breast returns to its pre-pregnant state, has been hypothesized to be one of the factors contributing to the incidence and aggressiveness of PABCs (Schedin 2006; O’Brien and Schedin 2009; Jindal et al. 2014). Involution shares characteristics with an inflammatory environment (Stein et al. 2004), which in turn is supportive of tumor growth and spread (Lyons et al. 2011). We previously reported a specific gene signature that is able to discriminate between nulliparous and parous normal breast, suggesting that parity increases inflammatory processes and at the same time imparts protective effects such as alterations in estrogen responsiveness that could be more durable (Asztalos et al. 2010). Here, we investigated the possibility of such genes playing a role in PABCs. We first noticed that tumors from the parous group, regardless of time lapse between pregnancy and diagnosis, presented with a higher grade compared to the tumors from the nulliparous group. This observation has been attributed to a diagnostic delay between occurrence of the presenting symptoms and the initiation of breast mass workup (Basaran et al. 2014). In fact, there have been several reports on an association between PABC and high grade tumors (Basaran et al. 2014; Langer et al. 2014; Murphy et al. 2012). However, all of these studies were conducted using tumors from patients whose cancer was diagnosed either during pregnancy or within 1 year of delivery. Our study is the first to show that this high grade feature remains associated with PABC up to 10 years after pregnancy, and is therefore less likely to be due to decreased ascertainment associated with the post-partum period.

Gene expression profiling of our samples revealed that the two parous subsets were homogenous in terms of their expression profile and were different from the nulliparous group as defined by the expression of eight genes (Table 2). This suggests that pregnancy-associated changes persist in the human breast tumors for as long as 5–10 years after delivery. When this eight-gene set was used to discriminate between nulliparous and parous tumors, we found that the nulliparous group was more homogenous in terms of their gene expression compared to their parous counterpart.

Another interesting finding of our study was that TNBCs occur more frequently in PABCs than in nulliparous cancers. In fact we only had one TNBC sample in the nulliparous group, which prevented us from including a nulliparous TNBC subgroup for gene comparison purposes. We found that the parous TNBC group differentially expressed a subset of 14 genes, five of which also showed differential expression between the parous cases and their nulliparous counterpart. Therefore we conclude that the major factor in gene expression differences associated with pregnancy is attributable to the TNBC subgroup of the PABCs. Most interesting is the finding that five of these genes (*TGFB3*, *ESR1*, *PGR*, *TIMP2*, *ERBB2*) are common to the gene set that distinguishes the normal parous breast from the normal nulliparous breast and six of these genes (*CXCL1*, *CXCL12*, *ERBB2*, *IL1A*, *MMP12*, *TIMP2*) are common to the inflammatory signature we previously reported (Asztalos et al. 2010). The expression of five genes (*TGFB3*, *ESR1*, *PGR*, *TIMP2*, *ERBB2*) is in the same direction in the parous normal breast and the pregnancy-associated TNBC with the exception of *TIMP2*. Three of these genes, *ESR1*, *PGR* and *ERBB2*, are associated with the protective effects of pregnancy in the normal breast whereas lack of expression of these genes defines the TNBC subtype. Nonetheless, the consistency of expression of a subset of genes found in both the normal parous breast and in parous TNBCs is a significant finding that may have implications for potential biomarkers of breast cancer risk. These data are consistent with the hypothesis that pregnancy creates a tissue microenvironment favorable for the development of TNBCs.

Our finding that PABCs show a higher incidence of the TNBC phenotype than the cancers of nulliparous women are in agreement with Pilewskie et al. (2012) who report that TNBCs are statistically more likely to occur in recent pregnancy associated (within 2 years) breast cancers than all other categories. In that study, TNBCs comprised 34 % of the total in the recent pregnancy group (0–2 years) compared to 11 % in the nulliparous group. Another recent study examined the frequency of TNBC in patients that included 31 patients diagnosed during pregnancy or within 1 year of delivery (Madaras et al. 2014) and also observed a higher frequency of TNBC compared to the nulliparous group. Our findings closely mirror those results, with TNBCs comprising 41 % in both pregnancy groups and only 5 % in the nulliparous group. To that end, this study presents new data that indicate TNBC risk can persist beyond 2 years after pregnancy. These findings are particularly important since the TNBC phenotype is associated with poor prognosis in numerous studies (Carey et al. 2006; Liedtke et al. 2008; Cheang et al. 2008; Nguyen et al. 2008; Tan et al. 2008). The relatively high frequency of TNBCs could contribute to the overall bad prognosis that characterizes tumors detected after pregnancy.

It is recognized that various molecular subtypes of breast cancers exist, characterized by distinct gene expression profiles (Perou et al. 2000). We were therefore not surprised to find that the TNBC subgroup showed a distinct gene expression set, among genes pre-selected to represent inflammation, ECM remodeling, angiogenesis. This signature includes 14 differentially expressed genes, four of which were consistently more highly expressed (*CXCL1*, *IL1A*, *IL8*, *MMP12*) and ten genes that were repressed in the PABC TNBCs. Of the four overexpressed genes, three are linked to inflammation (*CXCL1*, *IL1A*, *IL8*), suggesting that TNBCs exhibit a more inflammatory phenotype than other subtypes. Using Oncomine, we discovered that 11 of the 14 genes were previously reported to be associated with the TNBC subtype (*p* value = 0.05, twofold change), with *CXCL1* in the top 3 % and *MMP12* is in the top 1 % of genes associated with TNBC (Minn et al. 2005; Chin et al. 2006; Tabchy et al. 2010). Interestingly, three of the 14 genes (*IL1A*, *ELN* and *TIMP2*) to our knowledge have not previously been reported to be associated with the TNBC subtype.

CXCL1 was originally reported as a secreted cytokine by human melanoma cells and implicated in melanoma pathogenesis (Richmond and Thomas 1988). Subsequent work implicated *CXCL1* in breast, bladder, colon, and ovarian cancer (Minn et al. 2005; Yang et al. 2006; Kawanishi et al. 2008; Li et al. 2004). *CXCL1* is overexpressed in ERα negative breast cancers and high *CXCL1* levels are correlated with reduced relapse-free survival and metastasis (Bieche et al. 2007). IL1A is a secreted cytokine that promotes inflammatory processes and angiogenesis (Matsuo et al. 2009; Voronov et al. 2003). *IL1A* expression was found to correlate with poor differentiation and decreasing ERα expression in breast cancer (Singer et al. 2003). The expression of the neutrophil chemo-attractant inflammatory chemokine IL8 was shown to correlate with ERα negativity in breast cancers (Freund et al. 2004) and increased metastatic potential of various breast cancer cell lines (Bendre et al. 2002).

Of the genes expressed at lower levels in the TNBC group, several have been associated with favorable outcome. The association of *TGFB3* mRNA expression with breast cancer prognosis and PABC remains somewhat elusive, especially since TGFB3 is highly regulated at the post-translational level (Flanders and Wakefield 2009). However several clinical microarray datasets indicate that elevated *TGFB3* mRNA is associated with good outcome and is capable of predicting disease free survival in breast cancer (Flanders and Wakefield 2009; van’t Veer et al. 2002; van de Vijver et al. 2002). Therefore, lower levels in our parous TNBC cases are consistent with poor prognosis. IHC staining of some selected proteins on TMAs showed that there is a reasonable correlation between mRNA and protein level for some genes (ER, PR, HER2, CDH1), but not others (CXCL1, TGFB3).

In conclusion, in the present study we investigated the expression in PABCs of a selected set of genes previously demonstrated to differentiate benign breast tissue based on parity. We found that the TNBC phenotype occurs more frequently among PABCs, and to our knowledge, this study is the first to report the elevated prevalence of TNBCs and higher grade in PABC as long as 5–10 years post-pregnancy. Further, these TNBCs were largely responsible for the gene expression differences detected between PABCs and cancers of nulliparous women. Of particular interest is our identification of a subset of five genes whose expression is similarly altered in both the parous normal breast and in the TNBC subset of PABCs. The relatively small size of this study, especially the sparse number of nulliparous subjects with TNBC, precluded a strong statistical test of this hypothesis; thus larger studies will be required. A survival analysis comparing nulliparous patients and PABCs by IHC subtype would also be warranted.

## Additional files

10.1186/s40064-015-1512-7 Delta C_t_ values (housekeeping-gene adjusted gene expression).
10.1186/s40064-015-1512-7 Antibodies, incubation times, and dilutions used for IHC analysis.
10.1186/s40064-015-1512-7 Calculations used to determine H score for TMA analysis.
10.1186/s40064-015-1512-7 Correlation between protein and mRNA levels for selected markers.

## Abbreviations

ECM: extracellular matrix
ERα: estrogen receptor alpha
PR: progesterone receptor
PABC: pregnancy associated breast cancer
TNBC: triple negative breast cancer
IHC: immunohistochemistry
TMA: tissue microarray
