# Supplementary material for: High incidence of triple negative breast cancers following pregnancy and an associated gene expression signature
Source: Springerplus. 2015 Nov 19;4:710. doi: 10.1186/s40064-015-1512-7 (PMC4653130; doi:10.1186/s40064-015-1512-7)
Supplement: Supplementary file 2 — 10.1186/s40064-015-1512-7 Antibodies, incubation times, and dilutions used for IHC analysis. [file 40064_2015_1512_MOESM2_ESM.docx]

High incidence of triple negative breast cancers following pregnancy and the associated gene expression signature. Breast Cancer Research and Treatment

Szilard Asztalos^‡^, Thao N. Pham^‡^, Peter H. Gann, Meghan K. Hayes, Ryan Deaton, Elizabeth L. Wiley, Rajyasree Emmadi, Andre Kajdacsi-Balla, Nilanjana Banerji, William McDonald, Seema A. Khan, and Debra A. Tonetti

^‡^Equal contributors

**Corresponding author**: Debra A. Tonetti, Department of Biopharmaceutical Sciences, University of Illinois at Chicago, Chicago, IL, USA, dtonetti@uic.edu

**Additional File 2.** IHC details.

| Antigen | Pretreatment | Block | Primary antibody | Secondary antibody |
| --- | --- | --- | --- | --- |
| ER | Sodium citrate buffer pH 6.0 | Dako protein block 10 min | Clone SP2 Ventana 1:50 30 min | Dako Envision+ 30 min |
| PR | Sodium citrate buffer pH 6.0 | Dako protein block 10 min | Clone 1E2 Ventana 1:50 30 min | Dako Envision+ 30 min |
| HER2 | Sodium citrate buffer pH 6.0 | Dako protein block 10 min | Clone CB11 Novocastra 1:50 30 min | Dako Envision+ 30 min |
| CXCL1 | Sodium citrate buffer pH 6.0 | Dako protein block 10 min | NBP1-51188 Novus Biologicals 1:50 30 min | Dako Envision+ 30 min |
| ECAD | Sodium citrate buffer pH 6.0 | Dako protein block 10 min | 3195 Cell Signalling 1:50 30 min | Dako Envision+ 30 min |
| TGFB3 | Sodium citrate buffer pH 6.0 | Dako protein block 10 min | SC-83 Santa Cruz Biotech 1:50 30 min | Dako Envision+ 30 min |
