# Supplementary material for: High incidence of triple negative breast cancers following pregnancy and an associated gene expression signature
Source: Springerplus. 2015 Nov 19;4:710. doi: 10.1186/s40064-015-1512-7 (PMC4653130; doi:10.1186/s40064-015-1512-7)
Supplement: Supplementary file 3 — 10.1186/s40064-015-1512-7 Calculations used to determine H score for TMA analysis. [file 40064_2015_1512_MOESM3_ESM.doc]

High incidence of triple negative breast cancers following pregnancy and the associated gene expression signature. Breast Cancer Research and Treatment

Szilard Asztalos^‡^, Thao N. Pham^‡^, Peter H. Gann, Meghan K. Hayes, Ryan Deaton, Elizabeth L. Wiley, Rajyasree Emmadi, Andre Kajdacsi-Balla, Nilanjana Banerji, William McDonald, Seema A. Khan, and Debra A. Tonetti

^‡^Equal contributors

**Corresponding author**: Debra A. Tonetti, Department of Biopharmaceutical Sciences, University of Illinois at Chicago, Chicago, IL, USA, dtonetti@uic.edu

**Additional File 3.** Calculations used for H score.

*H score for Nuclear algorithm:*

H-score = (percent high intensity nuclei * 3) + (percent medium intensity nuclei * 2) + (percent low intensity nuclei * 1).

*H score for Membrane algorithm:*

H-score = (percent 3+ cells * 3) + (percent 2+ cells * 2) + (percent 1+ cells * 1).
