# Supplementary material for: High incidence of triple negative breast cancers following pregnancy and an associated gene expression signature
Source: Springerplus. 2015 Nov 19;4:710. doi: 10.1186/s40064-015-1512-7 (PMC4653130; doi:10.1186/s40064-015-1512-7)
Supplement: Supplementary file 4 — 10.1186/s40064-015-1512-7 Correlation between protein and mRNA levels for selected markers. [file 40064_2015_1512_MOESM4_ESM.docx]

High incidence of triple negative breast cancers following pregnancy and the associated gene expression signature. Breast Cancer Research and Treatment

Szilard Asztalos^‡^, Thao N. Pham^‡^, Peter H. Gann, Meghan K. Hayes, Ryan Deaton, Elizabeth L. Wiley, Rajyasree Emmadi, Andre Kajdacsi-Balla, Nilanjana Banerji, William McDonald, Seema A. Khan, and Debra A. Tonetti

^‡^Equal contributors

**Corresponding author**: Debra A. Tonetti, Department of Biopharmaceutical Sciences, University of Illinois at Chicago, Chicago, IL, USA, dtonetti@uic.edu

**Additional File 4.**  Correlation between protein and mRNA levels for selected markers.

| Marker | Parameter tested | | Correlation coefficient | p value |
| --- | --- | --- | --- | --- |
|  |  |  |  |  |
| ER | % 3+ nuclei | | 0.71 | <.0001 |
| PR | % 3+ nuclei | | 0.70 | <.0001 |
| HER2 | % 3+ membrane | | 0.79 | <.0001 |
| E-Cad | H score |  | 0.41 | 0.007 |
| TGFB3 | H score |  | -0.04 | 0.79 |
| CXCL1 | H score |  | 0.00 | 0.98 |
|  |  |  |  |  |
